# Albendazole Release from Silica-Chitosan Nanospheres. In Vitro Study on Cervix Cancer Cell Lines

**DOI:** 10.3390/polym13121945

**Published:** 2021-06-11

**Authors:** Daniela J. Hernández-Castillo, Erick Natividad de la Cruz Hernández, Dora M. Frías Márquez, Richard D. Tilley, Lucy Gloag, Patricia Quintana Owen, Rosendo López González, Mayra A. Alvarez Lemus

**Affiliations:** 1 Laboratorio de Nanotecnología-CICTAT, División Académica de Ingeniería y Arquitectura, Universidad Juárez Autónoma de Tabasco, Carr. Cunduacán-Jalpa de Méndez, Km 1 Cunduacán, Tabasco 86690, Mexico; djaznmin_07@hotmail.com (D.J.H.-C.); dorita78@hotmail.com (D.M.F.M.); rosendo.lopez@ujat.mx (R.L.G.); 2 Laboratorio de Epigenética, Universidad Juárez Autónoma de Tabasco, R/a. Sur 4ta Sección, Comalcalco, Tabasco 86650, Mexico; erick.delacruz@ujat.mx; 3 School of Chemistry, University of New South Wales, Sydney, NSW 2052, Australia; r.tilley@unsw.edu.au (R.D.T.); l.gloag@unsw.edu.au (L.G.); 4 Departamento de Física Aplicada, Centro de Investigación y de Estudios Avanzados del IPN Unidad Mérida, AP 73 Cordemx, Mérida 97310, Mexico; pquint@cinvestav.mx

**Keywords:** hybrid nanoparticles, albendazole, mesoporous silica

## Abstract

In this work, a pH-responsive drug-carrier based on chitosan-silica nanospheres was developed as a carrier for Albendazole (ABZ), a poorly water-soluble anthelmintic drug. Spherical silica nanoparticles were obtained by Stöber method and further etched to obtain mesoporous particles with sizes ranging from 350 to 400 nm. The specific BET area of nanoparticles increased from 15 m^2^/g to 150 m^2^/g for etched silica, which also exhibited a uniform pore size distribution. X-ray powder diffraction showed the presence of amorphous phase of silica and a low-intensity peak attributed to ABZ for the drug-loaded nanoparticles. A uniform layer of chitosan was obtained ranging from 10 to 15 nm in thickness due to the small concentration of chitosan used (0.45 mg of chitosan/mg of SiO_2_). The in vitro evaluation of hybrid nanoparticles was performed using four cervical cancer cell lines CaSki, HeLa, SiHa and C33A, showing a significant reduction in cell proliferation (>85%) after 72 h. Therefore, we confirmed the encapsulation and bioavailability of the drug, which was released in a controlled way, and the presence of chitosan delayed the release, which could be of interest for the development of prolonged release drug delivery systems.

## 1. Introduction

Hybrid nanomaterials have attracted attention due to the synergistic effect of the components. In nanomedicine, organic–inorganic nanomaterials are promising because of their demonstrated capability for enhancing biocompatibility, cellular uptake, and drug delivery. Polymers such as chitosan ((1,4)-2-Amino-2-desoxy-beta-D-glucan) exhibit suitable features for drug delivery, such as low cost, availability, non-toxicity, antimicrobial properties, tuneability and the advantage of facile combination with inorganic moieties by simple methods [1,2]. One of the main reasons for using chitosan in drug delivery is related to its pH-sensitive properties, because this characteristic allows the design of smart systems. Chitosan can be prepared as micro- and nanoparticles and as a coating, enabling the possibility to improve the properties of drug delivery systems and anticancer activity [3,4,5,6].

Silicon dioxide nanoparticles have also been explored in drug delivery, since the facility for obtaining SiO_2_ in a variety of shapes, sizes, surface composition and their ability to be easily attached to polymers such as polyethylene glycol (PEG), poly-lactic acid (PLGA) and chitosan (CS) for modifying their surface properties in biological applications.

Mesoporous silica (MSN) nanoparticles are attractive for the delivery of several active principles, and even biomolecules, allowing both intra- and extracellular delivery of proteins for improving their therapeutic effect, and protecting the biological cargo from the environment [7]. MSN has been successfully used for the delivery of anticancer drugs due to its low toxicity and surface area to volume ratio, which overcomes the disadvantages of some polymeric formulations, and because of its high stability, it can act as a protective shield of the active principles [8]. Silica nanoparticles can improve the solubility and bioavailability of drugs, low soluble drugs being of special interest. Due to this, the synergistic effect between chitosan and mesoporous silica nanoparticles in drug delivery has been evaluated for several anti-cancer drugs as methotrexate [9], 5-fluorouracil [10,11], doxorubicin [12] and curcumin [13], among others.

Albendazole (ABZ, Methyl [5-(propylthio)-1H-benzoimidazol-2-yl] carbamate) is an anthelmintic drug which can inhibit tubulin protein (responsible for cell multiplication), causes oxidative stress in cancer cells, and induces apoptosis [14]. Nevertheless, its low solubility in water (2 µg/mL) limits is use as a potential anticancer drug. Several attempts have been made to improve the solubility and efficiency of ABZ by using novel excipient formulations [15], new methods for preparation [16], and different micro and nano strategies for the delivery of this drug. For instance, micro-sponges made of Eudragit RS100 demonstrated high encapsulation efficiency and promising results in goats infected with *Haemonchus contortus*, whereas organic compounds have attracted more attention for the fabrication of nanocarriers to evaluate the antiproliferative effect of the drug [17,18].

Cervical cancer has the potential to develop malignancies, and even though there are treatments for this affection, its incidence among women is high, and there is a need to develop effective treatments with less adverse side effects than traditional ones [19]. Although the antiproliferative effect of ABZ has been evaluated in some types of cancer, to our knowledge, this drug has not been explored in cervical cancer cell lines. Thus, strategies to improve the treatment of this disease are needed, and nanotechnology is a promising tool for developing more efficient approaches. In this work, we synthesized and characterized hybrid mesoporous silica nanospheres coated with chitosan, as drug delivery systems for the release of albendazole. The antiproliferative effect of the nanoparticles was evaluated in four cervical human cancer cell lines: CaSki, HeLa, SiHa and C33-A. These results showed that ABZ drug was encapsulated inside the hybrid system made of chitosan-SiO_2_, and even at low doses, it exerts a cytotoxic effect against the cervical cancer cells used in this work, which enables its potential as an effective delivery strategy.

## 2. Materials and Methods

### 2.1. Reagents

Tetraethyl orthosilicate (TEOS, Sigma-Aldrich, St. Louis, MO, USA, 98%), hexadecyltrimethylammonium bromide (CTAB, Sigma-Aldrich, 99%), Methyl [5-(propylthio)-1H-benzoimidazol-2-yl] carbamate (ABZ, Sigma-Aldrich, 98%), methyl N-[(2S,3R,4R,5S,6R)-5-[(2S,3R,4R,5S,6R)-3-amino-5-[(2S,3R,4R,5S,6R)-3-amino-5-[(2S,3R,4R,5S,6R)-3-amino-5-[(2S,3R,4R,5S,6R)-3-amino-5-[(2S,3R,4R,5S,6R)-3-amino-5-[(2S,3R,4R,5S,6R)-3-amino-4,5-dihydroxy-6-(hydroxymethyl)oxan-2-yl]oxy-4-hydroxy-6-(hydroxymethyl)oxan-2-yl]oxy-4-hydroxy-6-(hydroxymethyl)oxan-2-yl]oxy-4-hydroxy-6-(hydroxymethyl)oxan-2-yl]oxy-4-hydroxy-6-(hydroxymethyl)oxan-2-yl]oxy-4-hydroxy-6-(hydroxymethyl)oxan-2-yl]oxy-2-[(2R,3S,4R,5R,6S)-5-amino-6-[(2R,3S,4R,5R,6R)-5-amino-4,6-dihydroxy-2-(hydroxymethyl)oxan-3-yl]oxy-4-hydroxy-2-(hydroxymethyl)oxan-3-yl]oxy-4-hydroxy-6-(hydroxymethyl)oxan-3-yl]carbamate (CS, Sigma-Aldrich, medium molecular weight), sodium hydroxide (NaOH, Sigma Aldrich, 97%), sodium carbonate (Na_2_CO_3_, Sigma-Aldrich, 99%), ammonium hydroxide (NH_4_OH, Meyer, Mexico City, México, 28–30%), dimethyl sulfoxide (DMSO, Sigma-Aldrich, 99%), Ethanol (C_2_H_6_O, Reproquifin, Ecatepec de Morelos, Mexico, 96%), and acetic acid (C_2_H_4_O_2_, JT Baker, Philipsburg, NJ, USA, 30%).

### 2.2. Synthesis of Mesopore SiO_2_ Nanospheres

Bare SiO_2_ was prepared by mixing 74 mL of ethanol, 20 mL of deionized water and 20 mL of NH_4_OH, then 3 mL of tetraethyl orthosilicate were added dropwise; after the TEOS was completely added, the mixture was kept under stirring for 1 h at room temperature. The suspension was centrifuged, and the precipitate was rinsed with ethanol and deionized water thrice. Then, the solid was dried at 60 °C during 4 h and then ground using an agate mortar with pestle. This sample was labeled as SiO_2_. Selective etching was achieved to promote mesoporosity in the particles through this methodology: 200 mg of SiO_2_ were suspended in 30 mL of deionized water and ultrasonicated for 30 min. The obtained suspension was poured into a solution consisting of 225 mg of hexadecyltrimethylammonium bromide, 45 mL of 1:1 (*v*/*v*) ethanol/water mixture and 0.875 mL of NH_4_OH and kept under continuous stirring at 750 rpm for 5 min. Then, 0.375 mL of TEOS were added dropwise and kept under stirring for 6 h. The precipitate was collected by centrifugation and washed with 10 mL of ethanol. The obtained suspension was dispersed in 30 mL of a 0.2 mol/L Na_2_CO_3_ solution under vigorous stirring at 50 °C for 8 h. The sample was centrifuged and washed with ethanol and water three times with 10 mL of ethanol and then three times with 10 mL of water; after this, the sample was dried at 60 °C and labeled as SiO_2_-MS.

### 2.3. Albendazole Loading and Chitosan Coating of Nanospheres

A 75-ppm solution of ABZ was prepared using DMSO as solvent, and 28 mL of this solution were taken and placed into a flask together with 500 mg of etched silica. The mixture was stirred for 4 h at 1000 rpm to occlude the drug into silica pores. Finally, SiO_2_-Albendazole particles were collected at room temperature by centrifugation (5000 rpm); after that, they were dried at 60 °C during 4 h. The sample was labeled as SiO_2_-MS-ABZ. Then, 300 mg of SiO_2_-MS-ABZ sample were dispersed in 236 mL of 0.08% *m*/*v* chitosan solution previously prepared (160 mg of chitosan were dissolved into 200 mL of acetic acid at 1% *v/v* and the pH was adjusted to 6 with a 1 M NaOH solution, to prevent microorganism’s formation) and stirred for 66 h. Nanoparticles were centrifuged and washed with 10 mL of deionized water twice and dried at room temperature. The samples will further be named as SiO_2_-MS-ABZ-CS.

### 2.4. Drug Release Profiles

Since chitosan is a pH sensitive polymer, two assays were performed for measuring the release of ABZ from silica: one using SiO_2_-MS-ABZ and SiO_2_-MS-ABZ-CS materials in only DMSO due to the solubility of the drug in this compound and because it is poorly soluble in other solvents, and the second one using SiO_2_-MS-ABZ-CS, adjusting the pH until acidic conditions were reached. For this, a wafer made by self-pressing 50 mg of each sample (in a 13 mm evacuable Pellet Press with a hydraulic press CrushIR-Pike Technologies, 3000 kg/m^2^) was placed together with 20 mL of DMSO inside a flask and kept under continuous stirring (70 rpm). At regular intervals of time, 3 mL aliquots of the supernatant were taken, and the absorption spectra were collected using a UV–Vis spectrophotometer (UV-2600, Shimadzu, Kyoto, Japan). The concentration of the drug was calculated by means of the corresponding calibration curve by following the increase of the absorption band at 309 nm. To reach an acidic pH for the SiO_2_-MS-ABZ-CS sample, a 0.1 M HCl solution was used, and the final pH was 4.8. To estimate the amount of drug encapsulated (Encapsulation Efficiency, EE%), Equation (1) was used; the data were obtained from measurements of the UV–Vis spectrum of the supernatant and interpolating in the calibration curve:

(1) $$EE\%=\frac{weight\;of\;the\;drug\;determined\;in\;the\;NP}{weight\;of\;the\;drug\;initially\;used}\;\left(100\right)\;$$

### 2.5. Characterization of the SiO_2_-Based Particles

The infrared spectra were collected from 4000 to 400 cm^−1^ using the ATR module of an FTIR Nicolet iS50 spectrometer (Thermo Fisher Scientific, Waltham, MA, USA). Hydrodynamic size and zeta potential were performed in a Zetasizer Nano Zs (Malvern, UK) with 175° optical arrangement. UV–Vis diffuse reflectance spectra of the solids were collected from 185 to 600 nm using a UV-2600 spectrophotometer (Shimadzu) at room temperature equipped with an integrating sphere and BaSO_4_ as reference (99% reflectance). The specific surface area, pore size distribution and fractal dimension were estimated from N_2_ adsorption–desorption isotherms at 77K measured in a Quanta Chrome Autosorb-iQ analyzer, the samples were degassed for 10 h at 70° C, while X-ray diffraction (XRD) measurements were recorded in a D8 Advanced diffractometer (Bruker, Billerica, MA, USA) with an X-Ray source of Cu (Kα = 1.5406 Å), 0.5 s per step and step size of 0.009°. Thermogravimetric analysis (TGA) was performed using a LabSyS Evo 1150 (Setaram, Caluire-et-Cuire, France) from 30 to 700 °C under Ar flow (20 psia) at a heating rate of 10 °C/min. The morphology of nanoparticles was observed using a JEOL field emission scanning electron microscope (FESEM) model JSM-7800F Prime and transmission electron FEI-Talos microscope model F200S at 200 kV with four in-column Super-X EDS detectors.

### 2.6. Antiproliferative Performance of SiO_2_-MS-ABZ-CS in Cervix Cell Lines

For each experiment, 3 × 10^3^ cells per well (CaSki, HeLa, SiHa and C33-A, that were obtained from ATCC and cultured following the supplier indications) were placed in 96-well culture plates, and incubated together with different doses of nanoparticles (1, 5, 10, 15, 20, 25 and 30 µg/mL); the effect of nanoparticles was evaluated after 72 h of incubation at 37 °C with 5% of CO_2_ in a humidified incubator. The cell viability assay was performed with crystal violet, according to the conventional procedures, then the optic density was measured using a microplate reader (BioTek, Winooski, VT, USA). Each experiment was performed by triplicate and cell proliferation was expressed as the average percentage of the three measurements.

## 3. Results and Discussion

### 3.1. Characterization of Nanoparticles

**Figure 1:**
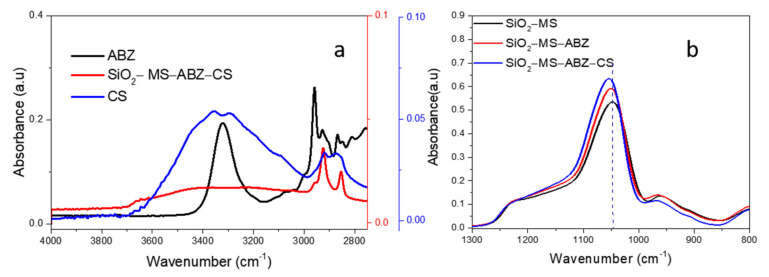
(**a**) High energy mid-FTIR region for ABZ, CS, and SiO_2_-MS-ABZ-CS, (**b**) low energy mid-FTIR region for SiO_2_-MS, SiO_2_-MS-ABZ, and SiO_2_-MS-CS.

Figure 1a shows the FTIR spectra for Albendazole, chitosan and SiO_2_ materials from 2000 to 500 cm^−1^ whereas in Figure 1b the 4000–2750 cm^−1^ region is displayed. The main characteristic bands for each of the components of SiO_2_-MS-ABZ-CS material cannot be fully identified due to the overlap of the signals; however, the presence of several weak bands in the 2000 to 1300 cm^−1^ region for this nanomaterial due to the presence of organic moieties [20] can be observed from Figure S1. However, by comparing to the ABZ spectrum, the spectrum of SiO_2_-MS-ABZ-CS exhibited similar peaks in the region between 3000 and 2700 cm^−1^ attributed to C-H vibrations, which appear slightly shifted toward lower frequencies (Figure 1a).

On the other hand, when the SiO_2_-MS-ABZ-CS spectrum is compared to those from ABZ and CS in the high energy region (Figure 1b), the contribution of both organic molecules can also be observed; the apparently weak intensity of these bands is attributed to the mode of measurement (ATR), since the signals exhibited at low energy are much more intense than those at higher wavenumbers. The most notable shift was the one corresponding to the band located around 1047 cm^−1^ attributed to stretching vibration of Si-O-Si bridging oxygen, which appears at 1051 cm^−1^ due to the interactions between atoms since the shift towards higher frequencies is related to an increase in the mass of the involved bonds. Finally, the signal at 796 cm^−1^ is attributed to the bending of the O-Si-O and Si-O-Si bonds.

**Figure 2:**
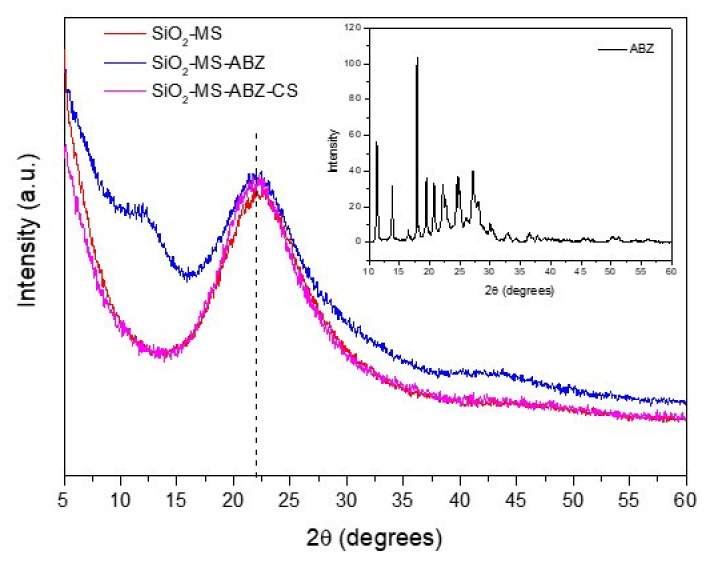
X-Ray powder diffraction patterns for the nanoparticles. Inset: pure Albendazole XRD pattern.

Amorphous SiO_2_ is structurally preferred for biological applications owing to its biocompatibility. In Figure 2, powder X-ray diffraction patterns of the prepared nanomaterials are shown, where the characteristic broad signal around 21.6° (2θ) corresponding to micro-amorphous silica can be observed [21]. This broad peak is related to the presence of surface hydroxyl groups and agrees with the PDF card 00-066-0178. When ABZ is incorporated with mesoporous silica (blue line), the broadening of the peak is clearly observed, in addition to a small signal appearing around 12°(2θ), which may be due to the presence of the drug, since the Albendazole pattern (Figure 2 inset) exhibited several peaks between 17 and 30° (2θ) that contributed to the broadening of the main shoulder of SiO_2_ in the XRD signal, and two relatively intense peaks below 15°(2θ) probably associated to the presence of both chitosan and Albendazole. Nevertheless, after chitosan coating, these features are no longer observed, mainly because chitosan exhibits a similar signal to amorphous silica around 20° and a small broad peak around 10°(2θ) [22]; after XRD and FTIR analysis, we can assume that ABZ was incorporated to the mesoporous silica, although complementary characterization techniques will help to confirm this.

**Figure 3:**
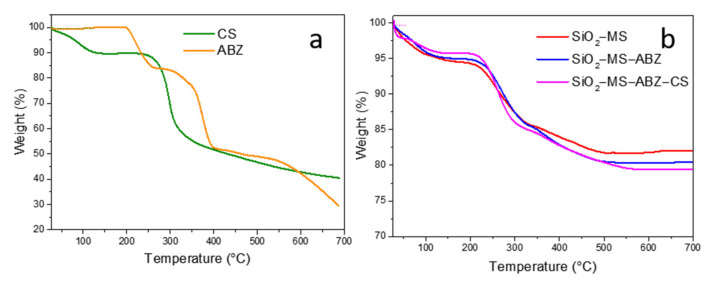
Thermograms of (**a**) pure chitosan and Albendazole, and (**b**) mesoporous silica, Albendazole-loaded mesoporous silica and Albendazole-loaded mesoporous silica coated with chitosan.

Thermogravimetric analysis was performed to evaluate the thermal stability of materials as well as to confirm the amount of drug and chitosan. Thermograms of chitosan and albendazole are shown in Figure 3a. For pure albendazole, a three-step curve was obtained; the first step took place at 199 °C, corresponding to a 16% weight loss related to the melting point of the drug, which occurs between 190 and 205 °C [23]; the second step (32%) at 291 °C may be attributed to the oxidation of the organic groups present in the molecule; the third loss (around 24%) took place between 402 °C and 700 °C, associated with the degradation of the organic material. On the other hand, chitosan showed two weight losses due to degradation processes of the compound; the first loss of mass (10%) took place between 30 and 130 °C, which corresponds to the removal of physically adsorbed water, since this compound strongly adsorbs water [24]; the second stage is attributed to the decomposition of the macromolecules, occurring between 240 and 700 °C [25].

Figure 3b shows the corresponding thermograms of SiO_2_-based samples. For the SiO_2_-MS sample, a gradual weight loss was observed from 30 to 100 °C and a second loss between 100 and 150 °C related to the evaporation of physical adsorbed water (ca. 5.6%). Between 150 °C and 320 °C, the sample lost almost 9% of its mass, followed by a less pronounced loss between 320 °C and 500 °C; the percentage of these two processes was around 12.5%, which is attributed to dihydroxylation of silica [26].

When ABZ was occluded in mesoporous silica and then coated with chitosan (SiO_2_-MS-ABZ-CS), the starting loss was about 5% wt., related to physical adsorbed water followed by a thermal stabilization between 100 and 200 °C. Above 200 °C and up to 500 °C both SiO_2_-MS-ABZ and SiO_2_-MS-ABZ-CS samples exhibited a similar behavior, where a shift in the slope of the curve can be observed around 320 °C, probably due to the melting and further oxidation of ABZ [27]. We observed that for the chitosan-coated particles, the temperature at which these processes take place was slightly shifted to approximately 300 °C.

**Figure 4:**
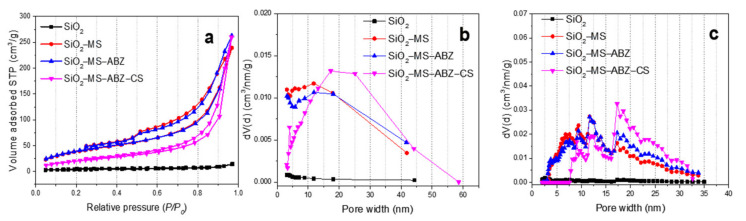
(**a**) N_2_ adsorption-desorption isotherms at 77 K of the unmodified and modified silica nanoparticles; (**b**) pore size distribution estimated by BJH method from adsorption branch (except for SiO_2_-MS-ABZ-CS sample, where desorption branch was used for calculations); (**c**) pore width estimated by NLDFT from adsorption branches and assuming cylindric pores.

To assess the differences after the etching process of the silica surface, N_2_ isotherms at 77 K were measured and are displayed in Figure 4. Regarding the adsorption process, we observed differences between pristine SiO_2_ and the rest of the samples. It has been reported that the Stöber method leads to the formation of microporous particles with poor BET areas, which is why additional treatments are used for modifying its textural properties. Here, the selective etching procedure was used to increase specific BET area by means of the generation of mesoporosity. For the SiO_2_ sample, we observed that N_2_ isotherm can be classified as type II with a very narrow hysteresis loop, commonly observed in mesoporous materials with some micropores. The low adsorbed volume at relative pressures below 0.3 indicates low specific BET area. By analyzing microporosity using the t-plot method (2 m^2^/g), we estimated that the mesoporous contribution to a specific BET area for the SiO_2_ was about 11 m^2^/g. Regarding the presence of pores in the SiO_2_ sample, it can be assumed that the number of pores is minimal, as confirmed by the small amount of adsorbed gas (Figure 4a). This value was increased after the etching procedure, as the slight increase in the adsorbed volume below 0.3 P/P_0_ values demonstrates. The SiO_2_-MS and SiO_2_-MS-ABZ samples exhibited same behavior for monolayer formation, resulting in very close SBET values (Table 1). This is related to the formation of mesopores after the surface erosion of the spheres through the etching process; the proximity of the BET area values for these samples can be interpreted assuming that the ABZ molecules are well dispersed on the surface of the spheres, and since the amount of drug is very low, there was no significant occlusion of the pores by the drug; thus, the BET area remains almost the same. However, the incorporation of chitosan changes this feature by decreasing the BET area value by more than 40% when compared to mesoporous samples. We assume that this probably happens because the chitosan molecules “seal” the pores, decreasing the available pores, resulting in a lower specific area. On the other hand, mesoporous silica samples exhibited type IV isotherms; for SiO_2_-MS and SiO_2_-MS-ABZ, the hysteresis loop closes around P/P_0_ = 0.4, and a small steep can be observed at the end of the desorption branch for both samples. This may be explained due to the cavitation phenomenon. The hysteresis loop is H4 type; the absence of the characteristic plateau at P/P_0_ ~ 1 indicates the presence of macropores or wider mesopores. Once again, the presence of chitosan on the surface of the silica particles modifies the hysteresis loop (SiO_2_-MS-ABZ-CS) to H1 type, showing that the capillary condensation takes place at higher relative pressures; this type of hysteresis is commonly observed in materials with a narrow range of mesopores. It is worth noting that the shape of the isotherm corresponding to the SiO_2_-MS-ABZ-CS sample is mainly due to the contribution of chitosan, but it is also important to remark that type IV shape is preserved, even after the incorporation of the polysaccharide. One of the preferred methods for reporting the pore size distribution is the BJH method, which has been widely accepted for mesoporous materials; nevertheless, due to the limitations of this method that can be found elsewhere in the literature [28] and when narrow mesoporous are present, NLDFT is the recommended method for pore size distribution calculations. As can be observed from Figure 4b, by estimating the pore size distribution with the BJH method, most of the pores ranged from 2 to 10 nm, by calculating the PSD through the NLDFT considering the sample as silica, assuming cylindric pores and taking data from adsorption branch (Figure 4c). For all the samples, we observed that the PSD curve provides us more information about pores between 3 and 10 nm. By comparison of the PSD plots, we observed that since the prepared materials were basically mesoporous, the range of the pore width obtained was between 3 and 45 nm (BJH) and 3 and 35 nm (NLDFT), which are very close to each other. In both methods, there is no contribution or detection of higher pores; thus, we can conclude that the absence of a final saturation plateau in the type IV isotherms is not related with the presence of macroporosity. Finally, in order to confirm the effect of the modification of SiO_2_, two additional parameters were obtained from physisorption data: the fractal dimension and C value from the BET equation. We observed that after the incorporation of chitosan, the C value was lower, whereas for unmodified silica, the C value was the highest. Since this parameter is related to the energy of adsorption in the first adsorbed layer of adsorbent, we can assume that both ABZ and CS decreases this energy, which is directly related to interactions between adsorbent and adsorbate. The fractal dimension was calculated using adsorption data and by comparing N-K vs. FHH models and the one with the higher correlation coefficient was reported in Table 1. Fractal dimension gives an approximation of the surface roughness, which could be one interesting feature to consider for interactions between nanoparticles and biological media. For the prepared materials, we confirmed that by inducing mesopores formation on the silica nanospheres, this parameter decreased to finally reach the lower value when the spheres were coated by chitosan. Since these values were around 2.5, this can imply that the surface is somewhat between bi-dimensional (smooth) and tri-dimensional (completely rough), which may facilitate the interactions with cells.

**Figure 5:**
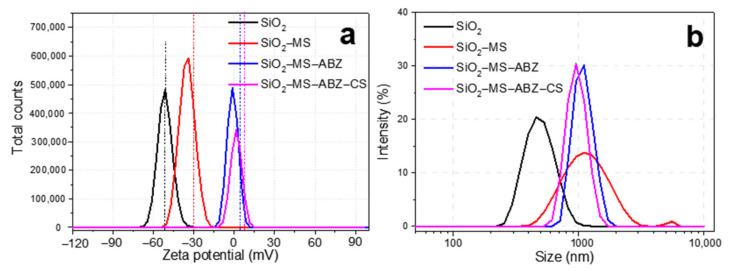
Analysis of (**a**) zeta potential and (**b**) hydrodynamic diameter of the silica-based particles.

DLS and ELS were used to measure the hydrodynamic diameter and zeta potential of the prepared samples, respectively. The results are shown in Figure 5, where it can be observed that after etching, the hydrodynamic diameter in water at pH 7 significantly increased from 400 nm to 1 µm (Figure 5a). The main difference among the samples was that the presence of organic molecules (ABZ and CS) on the surface of silica showed a narrower distribution than the other samples. When polysaccharides such as chitosan form multilayers, they exhibit aggregation as the concentration of the polysaccharide increases, mainly due to the presence of hydroxyl and amino groups [29]. This behavior was exhibited by our materials, since the formation of a chitosan layer over the mesoporous silica occurred, in addition to the presence of some agglomerates in the silica sample; the outcome is a significant increase of the hydrodynamic size of the etched particles (Figure 5b). In general, there could be an expected increase in size, depending on the concentration of chitosan used for covering nanoparticles, which may vary from a few 10s to several hundred, whereas the result in zeta potential is the obtainment of more electropositive particles after chitosan coating [30,31]. The logical explanation could be related to the porosity induced after etching of the surface, although additional studies are needed, since this effect still remains unclear [32]. Even though it is widely accepted that low values for the zeta potential tend to form unstable colloidal suspensions, we must consider that besides the zeta potential, factors such as the solution chemistry and properties of the materials might affect the stability of particles [33], in particular for chitosan when in contact to biological media, which could lead to stable suspensions when using culture media [34].

**Figure 6:**
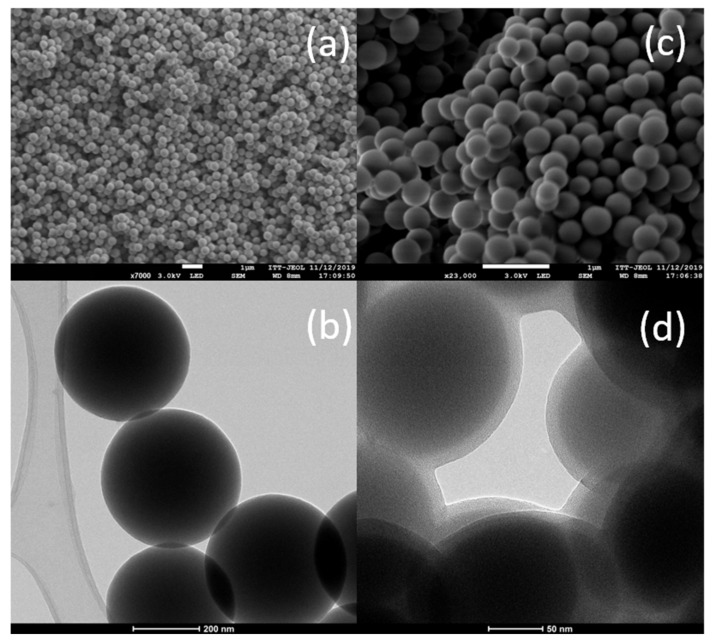
FESEM images (**a**,**c**) of bare silica nanospheres and HRTEM images (**b**,**d**) of mesoporous silica loaded with Albendazole and coated with chitosan.

The Stöber procedure has been widely used, since it produces spherical morphology, and depending on the reaction conditions, the particles obtained can be uniform in size. In Figure 6a, FESEM images of the SiO_2_ nanospheres used as starting material are depicted; there, we can observe that particles were homogenous in size, ranging from 350 to 400 nm (Figure 6b), forming agglomerates, where individual particles are clearly identified. By etching the surface of the spheres, a decrease in size is usually expected (Figure 6c). For the etched particles, we observed by HRTEM (Figure 6d) that the size slightly decreased for mesoporous chitosan-coated nanospheres and a homogeneous coating layer of about 10 to 15 nm encloses the surface of the nanoparticles.

### 3.2. Drug Release Test

**Figure 7:**
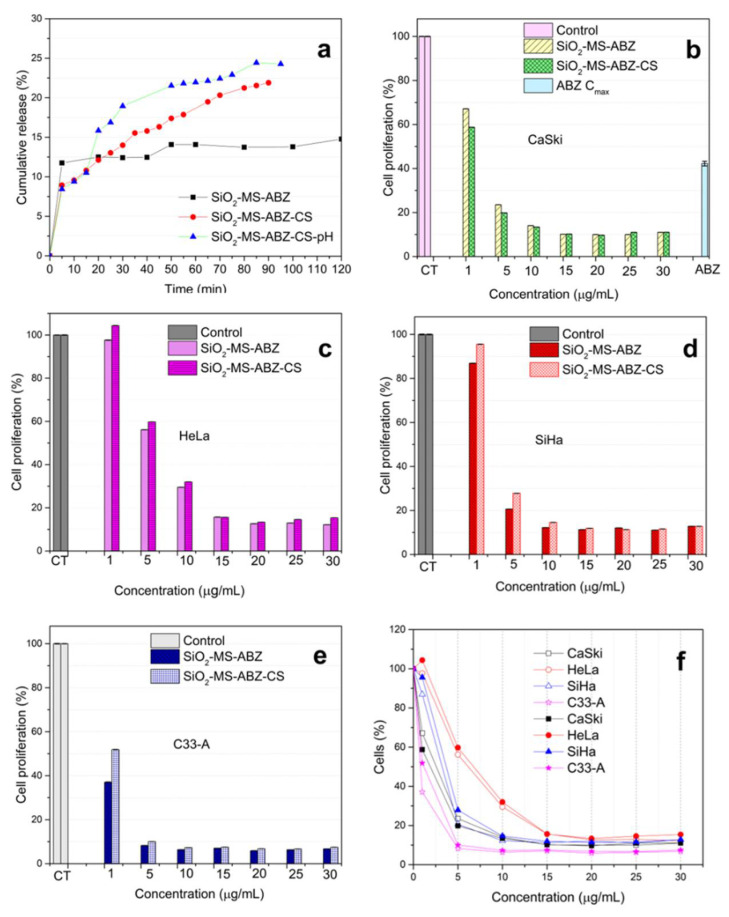
(**a**) Drug release profiles from uncoated and coated mesoporous silica; pH was adjusted with HCl until 4.8 value was reached (t = 30 min), and cell proliferation determined by crystal violet after 72 h of incubation with different doses of nanoparticles (**b**) CaSki, (**c**) HeLa, (**d**) SiHa and (**e**) C33-A cell lines. The percentages are the result of three replicates; the bars indicate estimated error. Additionally, (**f**) shows the comparison; (**b**–**e**) the hollow symbols represent the results obtained for uncoated particles, whereas the filled ones correspond to chitosan-coated materials.

One of the main advantages of the use of nanoparticles as vehicles is the potential for improving the properties of drugs such as chemical and thermal stability, solubility, and cellular uptake. In this research, we selected Albendazole since there exists some reports on their activity against cancer cell proliferation [14], but its low solubility in water limits its use. The drug encapsulation efficiency calculated was 28%. In Figure 7a, the drug release profiles for ABZ-loaded mesoporous particles are shown; the release assay was performed using DMSO as dispersant for both SiO_2_-MS-ABZ and SiO_2_-MS-ABZ-CS, whereas for SiO_2_-MS-ABZ-CS-pH, the release data displayed in the graph corresponds to the measurements performed also in DMSO but by adjusting pH. Since chitosan is pH-sensitive and its degradation takes place under acidic conditions, here, we compared the release of ABZ from coated silica particles with and without pH adjustment, mainly because cancer cells exhibit lower pH than neutral. The release from uncoated mesoporous spheres was also measured as a reference. From the graph, we observed that almost 12% of the ABZ was discharged from the SiO_2_-MS material during the first 5 min, but as time goes by, the amount of released Albendazole remains almost the same, and after 120 min, the total cumulative release of the drug was around 15%. In contrast, the release from SiO_2_-MS-ABZ-CS reached 22%, but in a more controlled fashion. For this material, the release of the drug could take place due to diffusion throughout the chitosan layer; however, when pH is modified, the amount of drug released increased. In the corresponding plot of Figure 7a (blue triangles), from t = 0 to t = 15 min, the pH of the suspension was around 11, and we observed that the drug released was lower than the released from uncoated silica. From t = 15 to t = 30 min, the pH was 6.7 and a slight increase in the release was observed, and when the pH finally reached 4.8, due to the solubilization of amino groups contribution in this sense to a double mechanism of release: diffusion and dissolution. Despite the low efficiency of encapsulation, we observed that almost all the drug was released and the improvement by using chitosan was clear. We can assume that perhaps the lower fractal coefficient of the SiO_2_-MS-ABZ-CS facilitates the liberation of the drug from the surface, which can also explain the behavior exhibited by the SiO_2_-MS-ABZ since the release from this material is slower, probably because the surface is less smooth and some ABZ molecules can remain trapped through their pathway to the surface.

### 3.3. In Vitro Assessment of Antiproliferative Effect

Crystal violet staining was performed after the nanoparticles were incubated during 72 h together with cells of the three different human cervix cancer cell lines: CaSki, HeLa, SiHa and C33-A (Figure 7b–e), and also with monocytes cells (Figure S2). As reference, for the CaSki experiment, the equivalent concentration of ABZ of the maximum theoretical load of the particles (474.8 nmol) was also evaluated. Immediately, the differences in the response among the four types of cells are clearly observed. For CaSki, cell proliferation decreases as the nanoparticle’s concentration increases. The SiO_2_-MS-ABZ showed a significant effect in cell proliferation even at the lowest dose of nanoparticles (1 µg/mL), while chitosan-coated nanoparticles exhibited a slightly higher effect. Nevertheless, above 15 µg/mL, the performance of both materials remained, with no significant changes. This behavior was modified for HeLa, SiHa and C33-A cells, where, at doses below 15 µg/mL, the chitosan-coated nanoparticles exhibited slightly lower activity. Only in HeLa cells, for the concentrations of nanoparticles above 15 µg/mL, the effect of chitosan over the mesoporous particles seemed to improve the antiproliferative effect of the drug. In spite the low encapsulation efficiency, the use of the nanoparticles as vehicles for the release of the drug produced a major effect compared to the free ABZ; this has been reported by other groups since the mechanism of action of the drug is through diffusion or active transportation by membrane proteins, but the mechanisms of nanoparticles are mainly through endocytosis [35] and the previous interactions between nanoparticles and proteins of the cell membrane which indirectly affect its permeability facilitating the access of the drug to the inside of the cell. In summary, the major sensitivity to ABZ was detected for CaSki cells; this is probably associated to differences in reactive oxygen species (ROS) produced by cells which have been demonstrated to play an important role in the response to chemotherapeutic agents, as published by Filippova et al. [36], where they evaluated the ROS sensitization/protection of cervical cancer cells and then administered Doxorubicin (DOX) or cisplatin at doses of 2 and 5 µM for 48 h. They found that by adding an agent, DL-buthionine-(S-R)-sulfoximine (BSO), it sensitizes CaSki and SiHa cells for ROS production, inducing a decrease in cell viability to ca. 22% for CaSki with DOX using the higher amount of BSO (0.2 µM). Here, we observed similar behavior regarding sensitivity, since CaSki reached the lowest values in cell viability, with a slightly higher effect. Besides the differences among the cells in the response to different chemotherapeutic drugs, we can attribute the significant decrease in cell proliferation to the textural properties of mesoporous silica, which are related to the roughness of the surface of silica enhancing the cellular uptake of nanoparticles [37], mainly when low doses are administered (Figures S3–S5).

## 4. Conclusions

Hybrid mesoporous silica-chitosan nanoparticles were obtained as delivery systems for a low water-soluble drug, Albendazole. When modifying SiO_2_ with alkaline etching, notable changes were observed in the textural properties of the materials. These changes were attributed to the effect of the surface interaction of the CTAB surfactant cations with the SiO_2_ silicate groups during the synthesis process, which also improved the pore volume for the modified material, which allowed the loading of the drug, and the chemical structure of the drug was preserved, as well as its antiproliferative effect. The modifications in the surface of the final hybrid nanocomposite improved the released of the active principle, probably due to differences in surface roughness appraised by the fractal dimension, and changes in zeta potential. The in vitro release of ABZ from the chitosan-silica nanocomposite proved not only its potential as an alternative for the solubility issues of the drug, but also the significant antiproliferative effect against human cervix cell lines. Our results are very promising since the amount of drug used in this work at the lower doses of nanoparticles is similar or even below the value most of the papers have reported for different drugs. Additional work should be performed to improve the synergy between chitosan and mesoporous silica.

## Figures and Tables

**Table 1:** Parameters of the nanoparticles estimated by dynamic light scattering, electrophoretic light scattering, and nitrogen physisorption.

Sample	Mean Size nm	Zeta Potential mV	S_BET_ m^2^/g	C from BET Equation	Pore Volume cm^3^/g	Fractal Dimension ^1^ D
SiO_2_	461	−51.3	11	183	0.021	2.70 *
SiO_2_-MS	1116	−33.7	144	44	0.352	2.51
SiO_2_-MS-ABZ	1106	−1.37	150	37	0.384	2.50
SiO_2_-MS-ABZ-CS	951	+1.61	80	26	0.367	2.34

^1^ Fractal dimension was obtained using Frenkel–Halsey–Hill (FHH), except for (*) where Neimark–Kieselev (N-K) method was used.

## Data Availability

Data available in a publicly accessible repository that does not issue DOIs. Publicly available datasets were analyzed in this study. This data can be found here: www.nanoujat.org (accessed on 2 June 2021).

## Supplementary Materials

The following are available online at https://www.mdpi.com/article/10.3390/polym13121945/s1, Figure S1: Full FTIR spectra of the samples, Figure S2: Cell viability assessed by XTT assay in monocytes at different doses of nanoparticles, after 72 h incubation, Figure S3: Crystal violet assay for cell proliferation in HeLa cells using different doses of nanoparticles, the amount of free ABZ used was equivalent to the nominal loading of the drug in the silica nanoparticles, Figure S4: Optical microscope images at 75X of HeLa cells at t = 0 with the different materials at the dose of 30 µg/mL, (before incubation), Figure S5: 96-well plate of HeLa cells after 72 h of incubation with the different nanomaterials and doses, each experiment was measured by triplicated.
