# Supplementary material for: Albendazole Release from Silica-Chitosan Nanospheres. In Vitro Study on Cervix Cancer Cell Lines
Source: Polymers (Basel). 2021 Jun 11;13(12):1945. doi: 10.3390/polym13121945 (PMC8230914; doi:10.3390/polym13121945)
Supplement: Supplementary file 1 [file polymers-13-01945-s001.zip › polymers-1232151-supplementary.pdf]

Supplementary Information

Albendazole release from silica-chitosan nanospheres. In vitro study on cervix cancer cell lines

Daniela J. Hernández-Castillo [1], Erick Natividad de la Cruz Hernández [2], Dora M. Frías Márquez [1], Richard D. Tilley [3], Lucy Gloag [3], Patricia Quintana Owen [4], Rosendo López González [1] and Mayra A. Alvarez Lemus [1,*]

[1] Laboratorio de Nanotecnología-CICTAT, División Académica de Ingeniería y Arquitectura, Universidad Juárez Autónoma de Tabasco. Carr. Cunduacán-Jalpa de Méndez, CP 86690, Km 1 Cunduacán, Tabasco, México.

[2] Laboratorio de epigenética. Universidad Juárez Autónoma de Tabasco, R/a. Sur 4ta sección, Comalcalco, Tabasco, México.

[3] School of Chemistry, University of New South Wales, Sydney, NSW 2052, Australia

[4] Departamento de Física Aplicada, Centro de Investigación y de Estudios Avanzados del IPN Unidad Mérida, AP 73 Cordemx, 97310 Mérida Yucatán, México

Correspondence: mayra.alvarez@ujat.mx

1. Full FTIR spectra of the samples

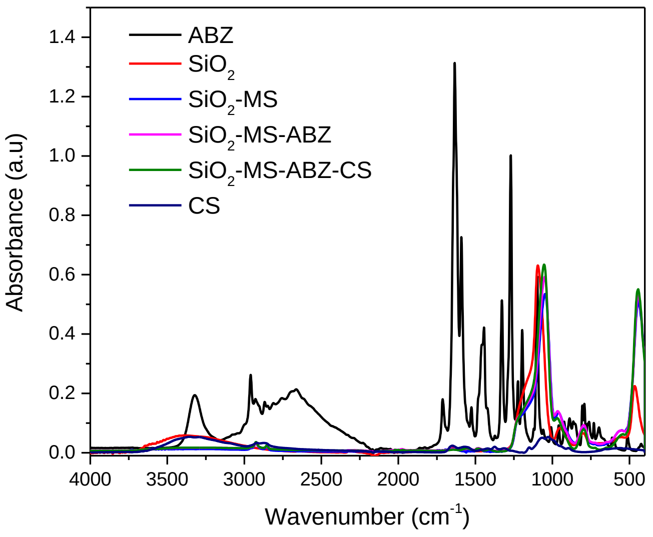

Figure S1. Full FTIR spectra of the samples

2. Additional experimental data

To evaluate the cytotoxicity of the nanoparticles at the different doses used in this work, monocytes cells were isolated form peripheral blood and incubated for 72 h. Cells were stained with XTT according to the specifications of the supplier (Roche)

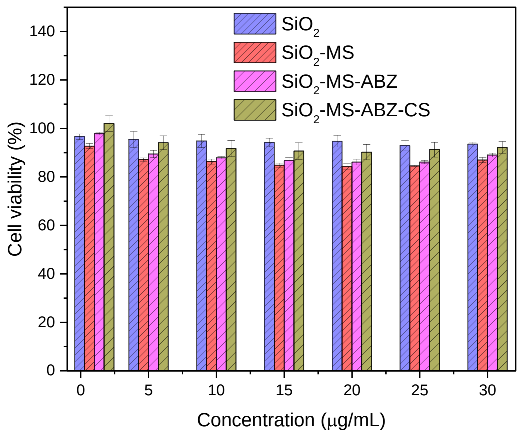

Figure S2. Cell viability assessed by XTT assay in monocytes at different doses of nanoparticles, after 72 h incubation.

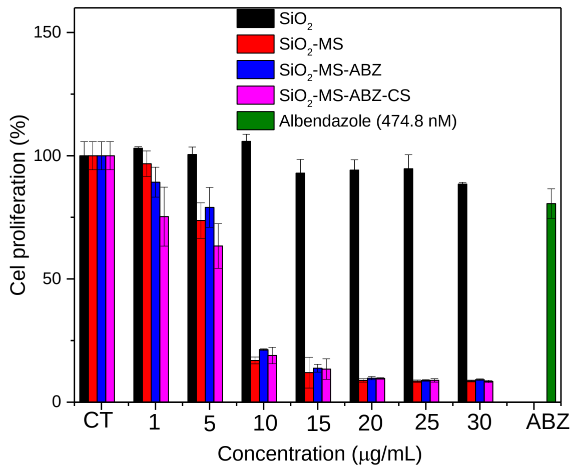

Figure S3. Crystal violet assay for cell proliferation in HeLa cells using different doses of nanoparticles, the amount of free ABZ used was equivalent to the nominal loading of the drug in the silica nanoparticles.

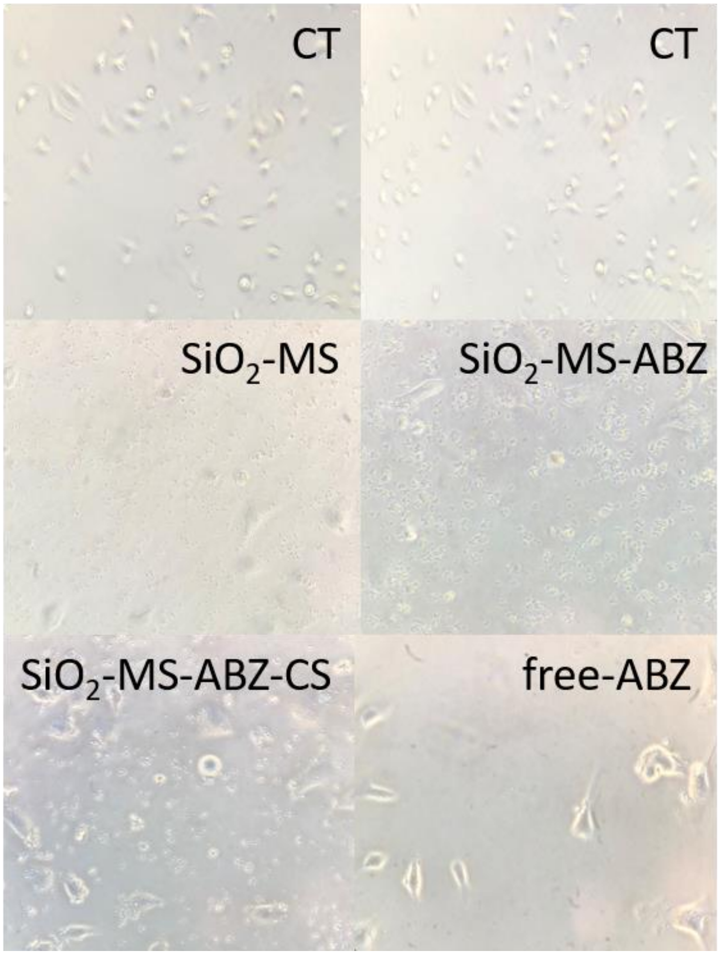

Figure S4. Optical microscope images at 75X of HeLa cells at t = 0 with the different materials at the dose of 30 μg/mL, (before incubation).

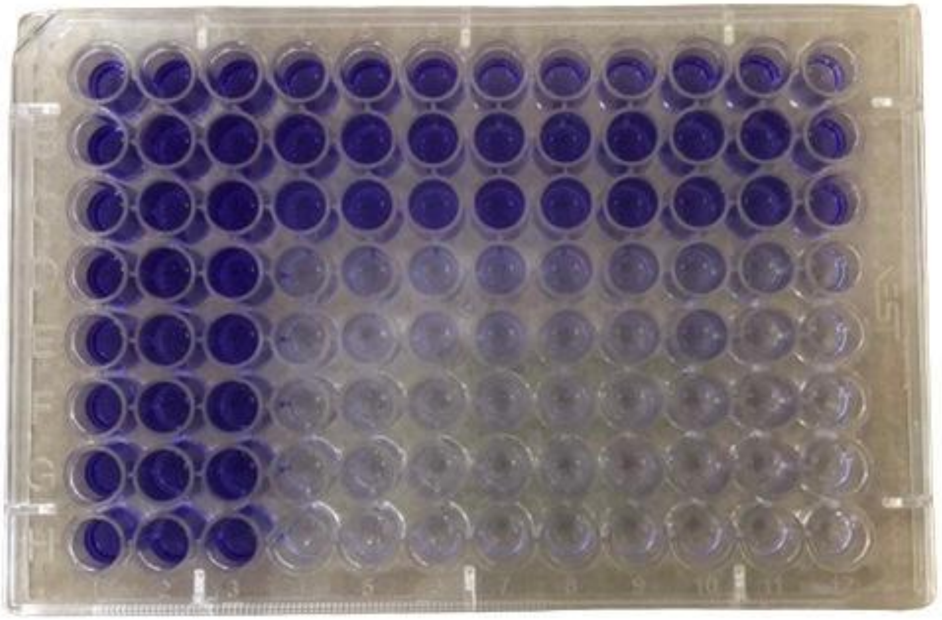

Figure S5. 96-well plate of HeLa cells after 72 h of incubation with the different nanomaterials and doses, each experiment was measured by triplicated.
